# Nothing less than a creative triumph: a study of children admitted to hospital for serious somatic illness or injury and their experience of art therapy

**DOI:** 10.3389/fpsyg.2024.1353507

**Published:** 2024-03-15

**Authors:** Patricia Loreskär, Per-Einar Binder

**Affiliations:** ^1^Department of Clinical Psychology, Faculty of Psychology, University of Bergen, Bergen, Norway; ^2^Educational and Psychological Counselling Service, Bergen Municipality, Bergen, Norway

**Keywords:** art therapy, children, hospital, observation, reflexive thematic analysis

## Abstract

**Background:**

Hospitalization can be an extremely distressing experience for seriously ill and injured children. Art therapy has a well-established clinical history, and recent research has begun to demonstrate its effectiveness in somatic pediatric wards. Descriptive and statistical research indicates that art therapy can alleviate anxiety and fear, improve mood, and enhance communication among children, parents, and healthcare professionals. This study aims to enhance our understanding of the specific elements of art therapy that facilitate a connection with the healthier aspects of the self.

**Method:**

A total of 12 hospitalized children, aged 4–12, participated in an art therapy session led by a certified art therapist. Verbalizations during the sessions were recorded, and subsequent observations were synchronized with verbatim transcriptions of audio recordings. A reflexive thematic analysis was conducted to identify relevant patterns of meaning.

**Findings:**

The study identified four significant dimensions of the children’s experiences during art therapy: (1) The child feels safe, (2) The child becomes a competent artist, bursting with creativity, (3) The healthy child emerges, and (4) The child achieves something beyond its limits – a triumph.

**Discussion:**

The active ingredients contributing to effective art therapy include the stimulation of creativity, guided art-making with scaffolding support, task variation, granting children control over the artistic process, encouragement of free expression, and the display of positive regard. This study also delves into the therapeutic alliance, emphasizing its role in facilitating children’s learning, self-expression, concentration, and the creation of work they take pride in. Additionally, certain psychotherapy-like interactions, such as control-mastery dynamics, were observed. Nevertheless, more extensive research with larger sample sizes is required to draw broader conclusions.

## 1 Introduction

The child does not answer and he begins coloring the sky with a block of blue beeswax. His mouth is hanging open. It seems like he wants to complete the painting. Suddenly he puts the block down and says: ‘Now I’m satisfied.’

This 7-year-old boy, tucked away in a hospital room, brushes strokes of vibrant color across a canvas. Amid an art therapy session, he radiates contentment and calm, a stark contrast to what we typically associate with hospitalized children facing severe health challenges. In this study, we will take a closer look at art therapy, specifically for little children who are hospitalized with severe somatic illness and injury and explore which elements of art therapy put them in contact with the healthy parts of themselves, and facilitate a sense of vitality and purpose, and thereby, strengthen their mental well-being.

Modern healthcare has made incredible strides in alleviating pain, mending injuries, and saving lives. But the path to recovery can be an emotional rollercoaster. Kids grappling with illness or injury often endure pain, discomfort, fatigue, and disrupted sleep (Jones et al., 2021). Their physical appearance can change dramatically, marked by scars, hair loss, and more. These young persons also contend with the unfamiliar hospital environment, missing out on time with loved ones and the joys of school life.

For the youngest patients with limited cognitive and emotional development, comprehending medical treatments can be bewildering (Boyd and Hunsberger, 1998). Fear and anxiety become companions on their journey (Rokach, 2016). Their self-esteem takes a hit, and their transformed appearance leaves them self-conscious, frustrated, and angry (Jones et al., 2021) Procedures involving needles and blood tests become sources of dread (Woodgate and Kristjanson, 1996; Kortesluoma and Nikkonen, 2006).

The toll extends to their parents, who play a vital role in supporting their children. These parents, witnessing their kids’ suffering, grapple with their own post-traumatic stress responses, including re-living traumatic moments, avoiding triggers, and heightened stress levels (Balluffi et al., 2004; Nelson and Gold, 2012). Hospital life also often clashes with the demands of home, work, and caring for other siblings (Foster et al., 2017).

Now, imagine the same 7-year-old boy immersed in the world of art therapy. Art therapy is a remarkable fusion of creativity, imagination, and emotional expression. It’s a mental health profession that blends psychology with visual arts, guided by trained art therapists (British Association of Art Therapists, 2023). Art therapy is employed across various contexts, including schools, healthcare clinics, and hospitals (Rubin, 2016; Shukla et al., 2022). The field lacks a universally accepted definition, encompassing a wide array of approaches. This particular study focuses on art production by child patients facilitated by a trained art therapist, with an emphasis on painting, drawing, and paper media art.

Art therapists work to create safe spaces for children to explore their feelings and unleash their creativity. Art therapy might have several goals that counter negative physical and psychological mechanisms inherent to medical treatment (Malchiodi, 2012, 2013):

1. Sense of control: When so much is uncertain, art therapy offers a sense of freedom and choice, allowing children to select materials, colors, and subjects, reinstating their sense of autonomy.
2. Self-Efficacy: Through their artwork, children visually manifest their capabilities, reminding themselves that they *can* accomplish things.
3. Communication: Drawing and painting become a unique language for children to express their thoughts, even when traditional talking therapies may be challenging.
4. Physical and Cognitive Rehabilitation: For those struggling with limited mobility, even the smallest brushstrokes can contribute to physical rehabilitation. Building concentration and endurance becomes an integral part of the artistic process.
5. Reducing symptoms, enhancing positive mood: Art therapy provides a temporary escape from illness, reducing pain, nausea, and fear. They can become ‘awakened’ to other and more pleasant experiences, thereby lifting moods and promoting relaxation.
6. Re-writing the illness narrative: Art therapy encourages children to create new stories, new identities, and new meanings amidst their challenging hospital experiences, shifting the child toward ‘posttraumatic growth,’ where the child creates a new life story, a new sense of self and creates meaning amongst distressing hospital experiences.

The field of art therapy, particularly for children in somatic settings, is still developing. While the domain lacks a clear-cut definition, various studies demonstrate its positive effects on children undergoing medical procedures (Reynolds et al., 2000; Abdulah and Abdulla, 2018; Shella, 2018). Inconsistencies in the field, such as varying outcome measures and the inclusion of art therapy within a broader scope of creative arts, make it challenging to isolate its specific impact. However, recent systematic reviews by Zhang et al. (2022), Clapp et al. (2019), and Aguilar (2017) confirm that art therapy benefits children hospitalized for severe illness and injury. These therapies help articulate thoughts, fears, and aspirations that are otherwise hard to express. Hyslop et al.’s (2018) study showed that art could be a tool for young children (aged 4–7) to express the emotional weight of symptoms and treatment. For example, one child’s drawing of a cannula attached to her hand helped initiate conversations about the illness and her feelings. Research by Massimo and Zarri (2006) reveals that drawings allow children to communicate their emotions. Their study, which focused on children diagnosed with cancer, showed that kids drew both their current state and their fears related to illness, thereby shedding light on their anxieties. Likewise, Woodgate et al. (2014) demonstrated that a drawing tool enabled children to express existential questions, providing another medium when words fell short. Drawing has also been shown to assist in coping and symptom management. Woodgate and Kristjanson (1996) conducted a unique study on children who had undergone surgery to understand their experiences of pain. Through participatory observation and semi-structured interviews based on drawings, they gleaned insights into the children’s understanding of pain and what could help alleviate it. The quantifiable impact of art therapy on reducing anxiety and depression has been validated in various studies, such as those by Gürcan and Atay Turan (2021), Altay et al. (2017), and Piasai et al. (2018). These studies used control groups and physiological measures to support their findings.

Art therapy may be a multifaceted tool for hospitalized children. It aids in communication, emotional well-being, and coping skills, thus making it a crucial aspect of integrative pediatric healthcare. While studies suggest art therapy benefits children in healthcare settings, many questions remain. Key concerns include identifying the elements that make art therapy effective and how children perceive its impact. Children often struggle to have their voices heard in medical contexts, where parents usually dominate conversations (Coyne and Kirwan, 2012). It is crucial to empower children to share their experiences, respecting their unique modes of expression like behavior, play, and drawing (Anning and Ring, 2004). With that in mind, the overarching aim of this study is to better understand children’s experience of art therapy based on their own expressions.

Given our need to add more understanding to what works, the purpose of this study is to investigate children’s experience of art therapy when seriously ill or injured. The purpose of this study is to identify commonality in experiences among hospitalized children undergoing art therapy without making distinctions based on the reasons for hospitalization or any personal characteristics of the participating children. The focus is on uncovering the specific aspects of the art therapy experience that are typical for this group rather than examining how different patient groups react to art therapy.

## 2 Methods

### 2.1 Study setting

While art therapy is common in mental health settings, it’s less so in somatic health. Haukeland University Hospital is an exception, offering art therapy to pediatric patients. It is one of the biggest hospitals in Norway, with approximately 4,000 children being admitted annually to the somatic and surgical departments of the Clinic for Children and Youth. Families learn about this option through nurses, the art therapist, or information in the wards, and sessions can occur in various hospital locations.

At Haukeland Hospital, art therapist Irén Kleiven offers patient-oriented, exploratory art therapy sessions (for stories from her work, see Kleiven, 2022). Families learn about this option through nurses, the art therapist, or information in the wards. Sessions are conducted in the patients’ rooms, though they may occasionally occur in makeshift settings such as hallways. These sessions are designed to be individualized, catering to one patient at a time, with the option for family members to participate at the child’s request. Within the context of this study, a few of the children had previous art therapy experience, while for most, it was a novel engagement. The art therapist is equipped with a trolley laden with various art supplies, including aquarelle paints, colored pencils, beeswax crayons, scissors, glue, and papers of diverse colors. Out of the 12 children participating in this study, 11 engaged in creating beeswax crayon drawings and aquarelle paintings. However, children were also encouraged to experiment with different art forms, with one child opting for mixed media.

Typically, in initial sessions, the art therapist invites the child to suggest a motif, which she then quickly sketches on a small paper using beeswax crayons, often choosing unconventional colors to highlight the limitless possibilities of drawing. The art therapist makes a point out of drawing things in surprising colors, like a pink sun or a golden car, and says ‘When you are drawing everything is possible.’ After, she paints aquarelle over, which does not mix with the crayons as the beeswax is fatty. She then tapes the borders of acid free, heavy paper to a board (since the water otherwise makes the paper wavy), and the child is free to begin their own creation. As the session progresses, the art therapist shows the children how to handle excess liquid, wash off unwanted pigment, mixing colors and ensuring that the areas they wanted filled with a solid color either could be filled with crayon or paint. The experience contrasts with the hospital’s medical atmosphere, aiming to let the child ‘lead the way.’ The sessions lasted between 25 and 60 min, with the youngest children typically painting for a shorter amount of time.

### 2.2 Recruitment and participants

During the data collection phase, the art therapist reached out to the head nurse of the pediatric ward to identify children who met the study’s inclusion criteria: children aged 3–12, proficient in Norwegian or English, and admitted for somatic care. Following this initial selection, the children and their families were directly approached to gauge their interest in participating in art therapy and to provide them with information about the study. Upon their consent, the nurse responsible for the child’s care was consulted on the session day to confirm the child’s treatment schedule and assess if their physical state allowed for participation. Data were gathered from the random first 12 children who fulfilled these criteria during the collection period, with the stipulation that only one data collection session would be conducted per day. The study’s cohort comprised 12 children, with ages ranging from 3 to 12 years (mean age of 7 years), and an equal distribution of boys and girls. All participants were native Norwegian speakers (Table 1).

**Table 1:** Overview of participants and sessions.

Gender	Age	Previous meetings with art therapist	Location	Participant	Materials	Duration
Girl	9	Multiple	Patient’s room	Individual	Mixed paper art	37 min
Girl	6	First	Patient’s room	Individual	Beeswax crayons, aquarelle	52 min
Boy	7	First	Patient’s room	Individual	Beeswax crayons, aquarelle	63 min
Boy	4	First	Patient’s room	Individual	Beeswax crayons, aquarelle	48 min
Girl	3	Multiple	Patient’s room	Individual	Beeswax crayons, aquarelle	31 min
Girl	11	First	Patient’s room	Individual	Beeswax crayons, aquarelle	58 min
Boy	7	First	Patient’s room	Individual	Beeswax crayons, aquarelle	57 min
Boy	10	First	Patient’s room	Family (child +1 parent)	Beeswax crayons, aquarelle	46 min
Boy	7	First	Patient’s room	Individual	Beeswax crayons, aquarelle	32 min
Girl	11	Multiple	Patient’s room	Individual	Beeswax crayons, aquarelle	24 min
Boy	4	First	Hallway	Individual	Beeswax crayons, aquarelle	25 min
Girl	12	First	Patient’s room	Individual	Beeswax crayons, aquarelle	49 min

### 2.3 Data collection

To study children’s experience of art therapy, the first author used digital audio recordings, observations, and post-session chats. Field notes focused on structural features, interactions, and activities. This data, recorded in real-time, emphasized the child’s experience, reactions, and choices. Observations were naturalistic and unstructured. The first author functioned as a ‘participant as observer,’ known but only engaging with the children post-session chats, offering what Van Maanen (1988) would chategorize as a realist account of the therapy experience. She based her field notes on Mulhall’s (2003) criteria: (1) structural and organizational features (e.g., the room, the set-up of the art therapy session, and art materials), (2) the people in the room and their behavior, interactions (e.g., actions and facial expressions), (3) process of activity (elements specific to the art therapy), and (4) verbalizations (e.g., self-talk, interjections, dialogue). Events were recorded chronologically, and the children’s behavior was noted side by side with the events and elements that preceded them (Green and Hill, 2005). To safeguard the validity of the data collection process, the art therapist was afforded the opportunity to review the transcriptions once the audio recordings were synchronized with the observational notes.

### 2.4 Data analysis

The transcriptions from the art therapy sessions were analyzed using reflexive thematic analysis, as outlined by Braun and Clarke (2022). It is realist, meaning it aims to capture people’s own perspective and remain recognizable to them, and to describe the reality as it was expressed in the dataset. At the same time, we have attempted to analyze the children’s experiences, at a more underlying or implicit level. The analysis involved five steps:

1. Familiarizing myself with the dataset. The transcripts were read and re-read and discussed by both authors. Brief notes was taken about analytical ideas related to individual data items, and the dataset as a whole. For example, during the data collection phase, an observation was made regarding a perceptible shift in the children’s demeanor when presented with multiple choices by the art therapist during the art therapy sessions. The children appeared to enjoy the opportunity to make choices, engaging in the selection process actively and with self-confidence. This observation led to reflection about the possible significance of agency within the context of art therapy.
2. Coding. The data was systematically worked through, and any segment that seemed relevant to the overall research question was labeled with a description. The first author collected and combined the coded labels, and then compiled relevant segments for each code. Examples of meaning codes were ‘proud of their painting,’ ‘adamant about finishing the painting’ and ‘drawing loved ones,’ and also – ‘children are given choices (paper size, colors, etc),’ ‘art therapist mirroring child’s language,’ ‘Art therapist verbalizing what she is doing.’
3. Generating initial themes. Based on the entire dataset, patterned core ideas were identified. Then all the coded data that seemed relevant to the candidate themes was collected and combined. Upon analyzing the aforementioned codes, it was discerned that the actions of the art therapist appeared to be strategically aimed at empowering the child with a sense of control. This was achieved through providing the child with choices, employing mirroring language that validated the child’s expressions were the ones that mattered, and verbally articulating her actions to ensure the child was aware of the ongoing process. Consequently, we aggregated all codes indicative of this pattern into a preliminary theme titled “Getting the child to feel in control.”
4. Developing and reviewing themes. In this phase of the work, the first author assessed the fit of the proposed themes, ensuring they covered the most important aspects of the data material, and that anything relevant to the theme had been included. Both authors discussed and considered the relationship between the themes, the wider context of the study, as well as what is known in the research field of art therapy and children in somatic pediatric wards. In deliberating our idea that the art therapist intentionally endeavored to afford the child a sense of control, we found alignment with existing research indicating that hospitalized children frequently experience fear and anxiety. Furthermore, the significance of analogous strategies in fostering psychotherapeutic relationships was acknowledged. This led to the conclusion that a sense of control might be a critical component for the efficacy of art therapy. As a result, we decided to retain the theme and began integrating it with other provisional themes. For instance, upon comparison with the code “Signalizing art therapy is part of the child’s world” we deduced that both themes likely contribute to engendering a sense of safety for the child.
5. Refining, defining and naming themes. The themes were described, based around a core concept or essence. Anything irrelevant was removed, and the themes was critically audited by the second author. A final, descriptive title was given to each theme. Upon synthesizing all themes indicative of efforts to ensure the child’s sense of safety, we organized these themes within a framework comprising four main themes, observing that they appeared to be interconnected, with each one facilitating the transition to the next. We posited that the art therapist’s incorporation of numerous elements designed to make the child feel safe had to do with helping them open up to creativity. This theme was denoted with a descriptive title, “Theme 1: The Child feels safe,” and we selected transcript excerpts that exemplified various ways the art therapist foster helped the child feel relaxed, seen and in control, thereby enhancing their feeling of safety.

#### 2.4.1 Reflexivity

Reflexivity is an integral part of thematic analysis Braun and Clarke (2022). The first author was, at the time the study was conducted, a student in the professional program in psychology who also had a master’s degree in political science. As an insider researcher with a background in psychology and political science, she focused on contextual factors, interactions, power dynamics, and communication. However, both authors are also outsiders, not being a part of the medical staff or trained in art therapy. The second author, the supervisor of the student researcher, is a clinical psychology professor with a child psychotherapy background. He especially focuses on emotional interaction, therapeutic alliance, and existential issues. This study recognizes the subjectivity and reflexivity involved in data collection and interpretation, emphasizing that context matters both at the individual and societal levels (Finlay and Gough, 2003).

An important aspect of reflexive thematic analysis is locating interpretation within the context. This means the data collected in this study should be understood within the framework of where it was produced, both closely around the child, as well as the larger societal setting. The societal setting was described at the beginning of this methods section and in the first part of the findings section.

### 2.5 Ethics statement

This study prioritized minimizing stress for hospitalized children and their families. Participation was voluntary, with informed consent and the option to withdraw at any time without affecting medical care. Parents were always present during art therapy sessions (or chose to leave on their own accord), and privacy and consent were carefully managed. Conversations with children were brief and sensitive to their condition, and all data was anonymized for privacy. The data was uploaded to a safe server (SAFE, University of Bergen) which no one outside the project (first and second author) had access. The project plan was approved by the Regional Committees for Medical and Health Research Ethics,^1^ and the Norwegian Agency for Shared Services in Education and Research Data AS^2^ as well as the hospital’s own ethics and review committee.^3^

### 2.6 Findings

#### 2.6.1 The stage – children and their parents in overwhelming circumstances

Entering the hospital environment was doubtless entering a particular setting. The families were staying in modern and bright rooms, but they were also sterile, cramped spaces, where medical equipment such as drip stands, bottles of chlorhexidine, crutches and fever thermometers mixed with puzzles, dolls and teddy bears. Everywhere were suitcases, backpacks, shoes and jackets, and on tables and window sills were plates, glasses and jugs of ice. Some shared the room with another family, divided by a simple screen. There were continuously unusual, invasive and frightening events, such as strangers entering the room, tense conversations and painful groans from behind the screen, alarms going off, sirens blaring outside and even helicopters landing and taking off right outside the window. Still, the therapist, the children and their families did not react, and never commented. Nurses continuously appeared and disappeared. They were all notably pleasant, positive and superficial. They always appeared with a knock on the door, but while they sometimes simply would pop their head in and then pop it back out again, their coming other times turned things upside down:

A nurse suddenly enters the room. ‘Hello, hello!’ she says with a loud voice and immediately takes command of the room. ‘Hello!’ says the therapist. ‘Excuse me for interrupting’ says the nurse to the child and therapist. ‘Yes, yes!’ says the therapist as if everything is in order. ‘Now I am going to ruin everything’ the nurse says half-jokingly, half-seriously, ‘changing everything around. You have to change to a different room.’ ‘Yes, these things happen’ the therapist says cooperatively. The child and mother say nothing. ‘Room 127’ says the nurse. (…) ‘Because this room we’re going to use for something else’ she adds firmly.

All children were seriously ill or injured. A majority was bedridden or sitting in a wheelchair. Only a few were able to sit on a chair for the art therapy session, and only one moved around freely. A few children demonstrated age-appropriate, childful behavior, with its typical hilarities:

‘Hi there!’ I say to the child, ‘What a lovely painting!’ The child gives me a long, silent look. He jumps up on the bicycle and zooms off.

However, many of the children were serious and quiet, and several had the behavior and speech of an older child. For these children, the age-appropriate childishness and playfulness was not present. Some had severely restricted movements of body parts, or the entire body. The children were markedly passive; almost all of whom the therapist first met in their rooms were looking at screens such as phones and iPads. They obeyed all instructions from medical staff and their parents. Many had still, expressionless faces. Some were openly fearful:

She [The child] has many tubes and cables connected to her body; they have been covered with a towel. (…) But suddenly the child bursts into tears and screams. The towel that hid the cables has fallen off. ‘Hide it–’ the child cries. ‘Just sit like that’ the mother says and tries to cover it with the towels. (…) ‘No! Hide it!’ the child screams.

Without exception, none of the children or their parents wanted to speak of the reason they were in hospital, or details of their stay. When asked, the parents would answer politely but shortly, or with hushed voices, shutting down or not inviting to further conversation.

It is now visible that the child’s hand is trembling. (…) ‘Is it both hands?’ the therapist asks the father carefully. The father nods, and closes his eyes.

In some of the chats the children would tell me about how their day had been, and in all these cases they told of medical procedures which had been difficult.

‘How has your day been?’ I ask the child. (…) ‘Before, when you were going to have your operation and stuff?’ the mother says. ‘Then I was afraid…’ the child says. He looks down.

Several had distorted body features, such as loss of hair, rashes, weight gain or swollen bodies or body parts. Some could not speak, for medical reasons or otherwise, and some made abnormal body sounds. In some hospital rooms there were strange smells. As a result, some initially avoided eye contact or turned their bodies away.

The children all had a parent with them. All parents were overly or superficially polite, and displayed cheerful and pleasing behavior toward the medical staff and the therapist. They tried to ensure cooperation, like this mother of a very reduced child:

It becomes quiet. The therapist looks at the mother. ‘What?’ the mother says. ‘I was just wondering if I could move the hat?’ the therapist says. ‘Yes, yes!’ the mother answers and waves a hand. ‘Hey, you have to answer, [Name]!’ she says to the child. The child doesn’t answer.

At the same time, many parents were quite affected. They appeared distant, sad and overwhelmed. Some displayed anger. Upon the art therapy session beginning, a majority immediately began looking at their phones. Some parents, who had met the art therapist before and knew what to expect from the session, chose to take a break outside the room. Others, who were more even-keeled, followed the art therapy session actively. Whenever the therapist asked a family member for assistance, for example fetching water or moving a bed, the family member delivered in a markedly eager manner. In some cases their willingness to perform a concrete task had to be curbed.

The therapist holds out a little bucket. ‘Now, I was wondering if Dad could get some water for me?’ she says. ‘Yes!’ the father says eagerly. ‘In this? Just this’ the therapist says, ‘Since I’m a bit boxed in here.’ The father springs into action and fetches water from the sink. (…) The therapist gives the plug for the hair dryer to the father. ‘Now, if Dad can plug this into the wall–’ she says. The father receives the cable and is so eager to assist that he knocks over a cup. He plugs the cable into the wall.

Some of the parents took pictures of their children drawing and painting. Some of these were parents of patients who seemed to be the most ill. During the art therapy session, many family members engaged in small talk around the painting and the materials. One parent painted together with her child. The parents invariably seemed pleased with their children’s creations and encouraged them to show or give them to others, including themself:

‘Maybe it’s a birthday present for me, since it’s my birthday today?’ says the father. (…) The therapist looks at the child. ‘Oh wow, is it Dad’s birthday today?’ she says happily. ‘Is it a birthday gift for me, [Name]? The beautiful painting?’ the father tries again, but the child doesn’t seem to listen. ‘Maybe?’ says the father.

### 2.7 Theme 1: the child feels safe

The first theme identified in this study concentrates on strategies the art therapist employs to establish a sense of safety for the children, a foundational aspect crucial for facilitating the subsequent art therapy process. Notably, for many children, the initial encounter with the art therapist, who wore a uniform similar to that of the nurses, was marked by hesitancy. The art therapist effectively addressed this challenge through several targeted actions. She introduced art therapy as something from the “world of children”; a place they know and master. She made them feel seen by repeating the children’s words and actions back to them, made them feel important and in control by letting them make all decisions, and explicitly commenting what she was doing and why. It seemed this had a clear effect; as the children relaxed the minds were at ease, allowing them to immerse into the creative process. Some of the children had met the art therapist before, and these children needed no special introduction. However, interestingly, over time the safe space seemed to have created a relationship where the these children felt safe to act out negative emotions. This theme includes observational notes that captures some of the art therapists concrete actions that built the safe environment, and at the end, some examples of the negative behavior that could play out.

The art therapy began from the moment the child saw the art therapist. The art therapist often pushed the art trolley into the room in front of her, such that the trolley was the first thing they saw. The art trolley was filled with brushes, colorful pigments, crayons, cutouts of whimsical painting and little toys. Recognizing elements from “their world,” the children relaxed and grew excited. Some children, however, the art therapist needed to win over by further emphasizing elements the child associates with their world. For example, the art therapist used a colorful, non-hospital apron to bridge the gap for a scared, 4-year old boy:

The child stands next to his mother. ‘Now let me show you!’ the therapist says. (…) The child doesn’t answer. (…) ‘Eh, uh’ the child whimpers. He makes crying sounds. (…) The child whimpers when the therapist turns her attention to him. ‘And here— Look what the carpenter made for me?’ the therapist says about the trolley. (…) She folds up the folding table on the side of the trolley. The child watches, but doesn’t look afraid. I put a chair next to the table. All the brushes and paint are now directly in front of the table and chair. When the child sees the setup he frees himself from his mother and stands next to her instead of holding her. (…) ‘Now… Look what I’ve got!’ the therapist says. (…) ‘The world’s prettiest painting apron!’ she sings. (…) The mother pulls the apron over the child’s head. The child now breaks completely free from his mother and eagerly sits on the stool.

To reduce any uncertainty, the therapist explicitly stated what she was doing, where she was going in the room and what was going to happen next. She made the child feel in control by constantly asking it to make small and big choices. She also continuously referred to the artistic process using ‘you’ or ‘us’ instead of ‘me’ or ‘I,’ thereby placing the child firmly in the center. The children were encouraged to make all critical decisions:

[The art therapist] lifts down the stand with the six glasses of water color pigment. ‘There’ the therapist says, ‘And now I’m looking forward to hearing– Do you want different colors? Or do you want one over the whole painting?’ ‘Mm’ the child says, ‘Want– some– different–’ ‘Ok’ the therapist says, ‘And what do you want to begin with?’ ‘Mm, blue’ the child says.

If the child did not answer when she asks them a question, she made a point out of waiting, or asking again:

The therapist dilutes the watercolor pigment to give it a lighter shade. (…) ‘Was the color nice?’ The child doesn’t answer. ‘Was it just right, or too light?’ the therapist says. There is a short silence. ‘Just right’ the child says. ‘Just right. All right’ the therapist says.

If she needed to show the child how to do something, e.g., spreading out excess paint to avoid puddles, the therapist held her hand over the child’s rather than holding the brush by herself. She often verbally emphasized that she wasn’t ‘permitted’ to do anything on the child’s painting, and if they asked her to draw something for them she would jokingly say something like ‘Do not look at me!’ If they asked her to make choices for them she always said ‘That’s up to you!’ The therapist mirrored the child’s language continuously, ensuring they felt ‘seen’:

‘There!’ the child says. ‘There!’ the therapist says encouraging. ‘Look!’ the child says. ‘Now look at that!’ the therapist says warmly. The child continues [painting]. ‘Oops!’ says the child when she gets more paint with her brush and a drop falls on the painting board. ‘Oops’ the therapist says, ‘Don’t worry. We can wash it off later. Just use some soap and water, right?’

Some children had an attitude and manner that unexpectedly came across as rude. One child bossed the therapist around, and also had down-putting comments. This child had a long history of coming to the hospital, and also having art therapy:

‘Should I erase it?’ the therapist asks. ‘Uh…’ the child says while thinking, ‘Yes.’ The therapist erases carefully. ‘Well, it’s not like you needed to be *that* careful, did you now?’ the child says reproachfully.

‘Should we cut it off, is that what you want?’ the therapist asks. ‘No’ the child says condescendingly, ‘That is *not* what I am saying.’ ‘No’ the therapist says, accepting. She measures: ‘Two point four’ she says and looks at the child. ‘*No*’ says the child, ‘Two point three.’ ‘Two point three, all right’ the therapist says.

Another child handled the materials roughly, like this child who has had several successful sessions of art therapy before, but this day is very reduced:

‘Is it this way you would like [the paper], or would you like it this way?’ says the therapist. The child is motionless. A few moments pass by. ‘It’s your decision,’ the therapist says with a soft voice. The child suddenly strikes the paper, and it flings to the side.

Once the child had held the ‘upper hand’ for a while, the child would drop the bullying attitude and the remainder of the art therapy session was characterized by warmth and collaboration. The therapist met all these odd or noteworthy behaviors with the same calm and cordiality. She acknowledges and validates the child’s statements, while not taking herself seriously or ceremonially:

‘There, let us glue it all together. Is it me who should glue, or you?’ the therapist says. ‘Uh, you’ the child says. ‘It’s always the *apprentice* who does it’ she adds. ‘So the apprentice… That’s what I was going to say’ the therapist answers, ‘I’m a bit like your assistant. Or apprentice.’ She begins to glue.

### 2.8 Theme 2: the child becomes a competent artist, bursting with creativity

Feeling safe and in control, the children became receptive to the art therapy process. The art therapist employed specific interventions to empower the children as proficient creators, demonstrating the use of beeswax crayons and aquarelle paints while expressing confidence in their abilities. The therapist’s support was instrumental in enabling the children to effectively complete their artwork, offering “invisible” assistance to maintain their focus. This assistance was provided through verbal acknowledgment of their actions and by promptly supplying crayons, paint, and water or suggesting task shifts without disrupting the flow of creativity. Moreover, incorporating elements with dramatic effects captivated and amused the children, enhancing their engagement. The art therapist seemed to ‘read’ the child – anything from a sigh or a yawn, the child putting down a paint brush or stretching for a color was noted - and adjusted the session accordingly. The child and therapist often ended up in a silent, close-knit cooperation with their hands working side by side. They clearly had a shared goal. In this enabling environment, the children seemed to not only experience creativity, but were also able to complete the painting.

In this theme, there are snippets that capture the way the child was made to feel able to create, and letting creativity flow. The following excerpt, for example, shows how the art therapist followed the child’s every move, and assisted it in such a way that moving forward was easy:

The child, who got the paint brush while the therapist was explaining how to dry excess liquid, begins immediately diluting the pigment. He shifts between diluting and soaking up, without saying anything. He reaches out for paper or clean water, which the therapist provides, back and forth. ‘There’ the therapist says, ‘Should we go over the sun, as well?’

This reading of the child also helped the therapist keep the child focused on the work until it was completed. One child began to play once the painting was completed, leaving the drying process to his mother and therapist:

‘Now it’s us adults left here’ the therapist says and laughs a little. ‘Don’t worry’ the mother says, ‘This is longer– This is the longest he’s *ever* sat like this.’ She waves with her hand. ‘I mean, he wouldn’t even have sat this long before.’ She pauses. ‘I mean, when he was *healthy*’ she adds.

The therapist consulted them on preferences in colors, paintbrushes, paper sizes and techniques. She taught them tricks and gratefully received tricks from them:

[In this art therapy session, the child has made a pop-up card.] ‘There. Now I’m going to show you something neat’ the child says, ‘Look at this!’ She opens the papers, and a blue square in the middle pops out. (…) ‘Ah!’ the therapist says and lets out an impressed sigh, ‘All the things I get to learn!’ She looks at the mother. ‘I’m so lucky!’ She laughs. At this, the mother also begins to laugh.

What is it like to feel *creative*? The children see the therapist introduce the technique using beeswax and water colors. They watch her with fascination, and afterwards, without fail, they wanted to get going. They grab for materials:

‘Can I, uh, draw… now?’ the child asks and tries to take the crayon from the therapist’s hand. ‘Yes, now I need to let you begin,’ the therapist says, ‘Now you need to begin.’ The child immediately starts looking through the crayons on the table and looks like he is going to start drawing. ‘Oh, wait!’ the therapist suddenly says. [The therapist makes the child choose a paper and which way it should be positioned.] ‘That way’ the child says about the vertical position, and immediately tries to begin drawing. (…) ‘Just a moment! A little moment!’ the therapist says as she tapes the paper to the painting board, ‘Soon you can begin!’ ‘Oh my god!’ the mother says dejectedly and laughs. (…) As soon as the art therapy removes her hands the child begins to draw. He begins with a yellow crayon in the middle of the paper. He is concentrated and seems determined.

Several end up drawing something that relates to the first image they ask the therapist to draw and paint. Without failure, the children believe they can master the technique. They all set out to use both beeswax crayons and aquarelle watercolor, even children as young as 3 and 4 years old. The therapist often refers to the white crayon as ‘magical,’ and after seeing how it stands out after she paints, the children keenly use the white too. She makes a point out of drawing things in surprising colors, like a pink sun or a golden car, because she says ‘When you are drawing everything is possible.’ The children then begin drawing with the same whimsical choices. The therapist avoided asking questions such as ‘what is this supposed to be?’ or encouraging them in any direction.

**Figure 1:**
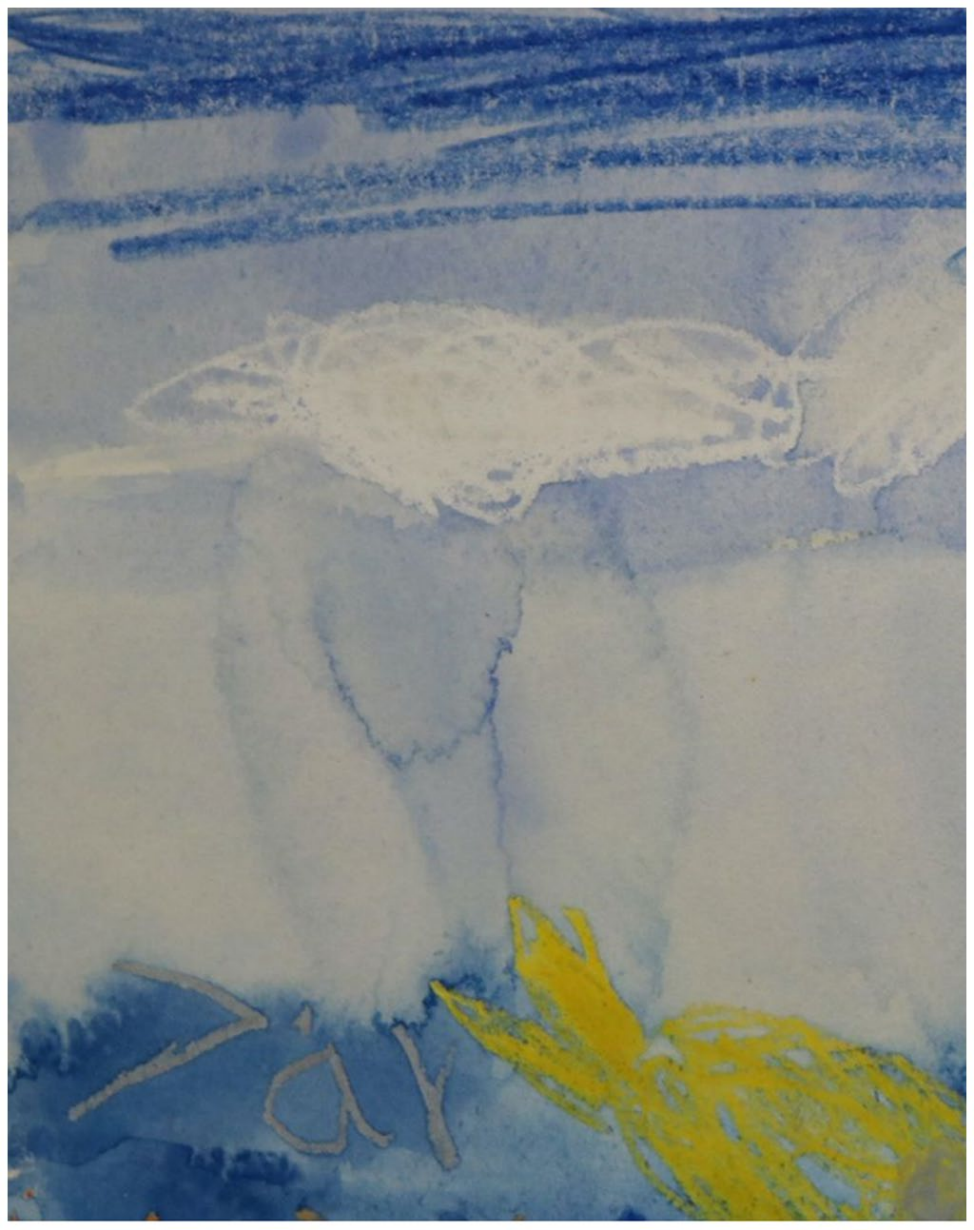
Detail from a painting by a 7-year old boy, where what at first was unintentional build-ups of excess water, instead of being removed was embraced as ‘rain on the horizon.’

They learn details of the techniques, such as taping the paper to the board (since the water otherwise makes the paper wavy), handling excess liquid, washing off unwanted pigment, mixing colors and ensuring that the areas they wanted filled with a solid color either could be filled with crayon or paint. Because they use aquarelle and water, the material is itself ‘alive’ and unexpected things happen to the painting, something the children engaged with or even learnt to use creatively, like a 7-year old boy who had excess water below a cloud he had drawn, and instead of painting over it to create a smooth sky, let it look like a cloud dropping rain in the distance (see Figure 1), or a 12-year old girl who learnt that by adding water that trickles on the painting a clear trail is created, and used this to create a faint teardrop on an eye.

It seemed that while the children enjoyed drawing, they enjoyed the painting even more. The water color quickly had a new and dramatic effect on the drawing, emphasizing colors and contrasts. The children painted with great concentration:

The child begins to paint. (…) He is careful about the edges. He frowns. (…) The child is sitting with a straighter back. He is painting with his mouth open. His cheeks are completely relaxed.

At the end of the session they would pull the tape off, revealing a clear border, almost looking like a passepartout. The children seemed to thoroughly enjoy this part of the process:

‘There’s one thing left that you’re going to get to do, you’re going to–’ the therapist begins. ‘Pull off the tape!’ the mother guesses, ‘How fun!’ The child is eager and reaches his hands out. (…) The child pulls the tape off. ‘Nice’ the therapist says, ‘That was the first one.’ She begins picking the corner of the next tape. ‘I want the next one too,’ the child says. (…) The child pulls off the second tape. He gives the tape to his mother. He is eager to begin with the next. ‘Can I pull this one?’ he says and looks at the therapist.

Talking about the materials and techniques became a ‘safe’ platform for both children and parents and a harmless way to engage in conversation.

### 2.9 Theme 3: the healthy child emerges

Once the child had become safe and relaxed, the art therapist the art therapist effectively nurtured the children’s sense of competence, leading to a notable surge in creativity. Through focused engagement and direct support, the children were encouraged to remain dedicated to their tasks until completion. This phase marked a transformative period where the healthy inner child emerged. Initially, most of the children entered the art therapy sessions exhibiting reserve, passivity, and evident signs of pain and discomfort. However, as they engaged in painting, their focus diverted from their ailments, igniting a transition to a state of excitement, activity, and increased mobility. There was a stark contrast between their physical exterior, and the painting that emerges. One may never have thought it was painted by someone so ill or badly injured - it seemed the children’s conditions did not set any limits on them. It seemed as if the healthy self within the child was allowed to emerge, and the “patient” part of the child took a back seat.

Especially the younger children had a greater shift in mood, because they generally started out more hesitant, like this 7-year old:

He gives the therapist a flat, but not unfriendly look. The child almost seems startled. He is very quiet, and doesn’t move, but watches the therapist attentively.

The child gets going, and paints with long, self-confident strokes. (…) He dips the brush in paint and continues painting without hesitation.

In some cases the child was visibly reduced. In one instance, the therapist was informed that the child recently had been through an intense medical procedure. Naturally, the adults kept asking ‘Are you sure you want to paint?,’ to which the child insistently responded yes:

The girl is in a wheelchair, sitting completely still. She looks down, and almost seems absent. She is very passive. She doesn’t have any hair, except for a little dot on one side. (…) ‘It’s not long since she woke up from anesthesia,’ the mother says. ‘Yes’ the therapist says. ‘But she wanted– I asked her,’ the mother says, “Do you want to do it today?” “Yes” she said.’ ‘Yes’ the therapist answers.

There were many cases where the child was in visible discomfort, but the discomfort seemed to lessen while they were painting, like this 7-year old boy:

(…) In his left elbow a piece of cotton has been taped onto him. [The table is placed over him in the bed.] He keeps the left arm in a stiff, unnatural position, and keeps it under the table. The right arm has natural movements and he places this hand on the table. (…) He draws with his right hand while the left arm is still in a strange, stiff position under the table. (…) He tries different ways to draw and drag the crayon, and suddenly his left arm comes up on the table. (…) He holds the paper down with his left arm.

The same was seen in a 4-year old boy:

He has a cannula on his right hand and a wrist supporter with a blue stretchy bandage over, such that most of the cannula isn’t visible and can’t be moved. He also has a bandage on one of the fingertips. (…) The child first lifts his right hand, but seems reluctant to use it. He lifts his left hand, but hesitates. ‘Can you draw with one hand?’ the mother says. The child stretches his left hand out. ‘Yes, you can start with the left hand a little’ the mother says. ‘Sure, it’ll be fine!’ the therapist says, ‘That’s what’s so good about drawing, you can do it with both the left and right hand.’ The child begins doodling with the left hand. He becomes increasingly excited, and begins supporting the left hand with the right. He draws with both hands holding the crayon. (…) The child takes many colors. Suddenly he takes the crayon with his right hand. He begins drawing intensely with a black crayon. He draws a little bit with his left, then changes back to the right. ‘Tu tu tu’ he hums. He draws many lines, figures and scribbles in different colors across the paper.

One 7-year old boy went from not wanting to draw because he was in pain, to being so eager he could not wait for his own paper:

The child is lying in bed (…). He has cannulas taped onto both hands. He doesn’t move his legs. (…) The child lies down and partly turns away from the therapist, so even though the table has been placed in front of him in bed he doesn’t seem ready to draw. ‘Is it possible for you to sit up, or….?’ the therapist says. (…) the child suddenly interrupts her by yelling angrily: ‘It hurts, and stuff!’ (…) When the therapist places the painting board on the child’s table he immediately sits up and looks at it. (…) ‘I’m going to draw’ the child says firmly. (…) He starts drawing on the therapist’s paper, first doodling in a corner, then drawing a figure that looks like it is flying or moving.

If the child is reminded of their illness or injury, however, the spell is broken:

‘Yes well, we’ve been a bit– almost haven’t slept this week. Right?’ the mother says to the child, ‘You’ve been really, really sick? You’re very tired now, aren’t you? That’s ok.’ When the mother says this the child begins to whimper and suddenly looks drained of energy. Suddenly it seems nobody knows what to say and the room becomes quiet.

Many of the children said that they thought it was fun to paint, but they were not able to go beyond this and explain what it was that made it fun, or how it made them feel. When chatting with the children, I asked them what they were thinking about while they were painting. Some said they did not know or did not answer, while two seemed to say their mind was resting for a short bit:

‘What were you thinking about while you were painting?’ I ask. ‘Uh…’ the child says, thinking, ‘I thought about *nothing*.’

**Figure 2:**
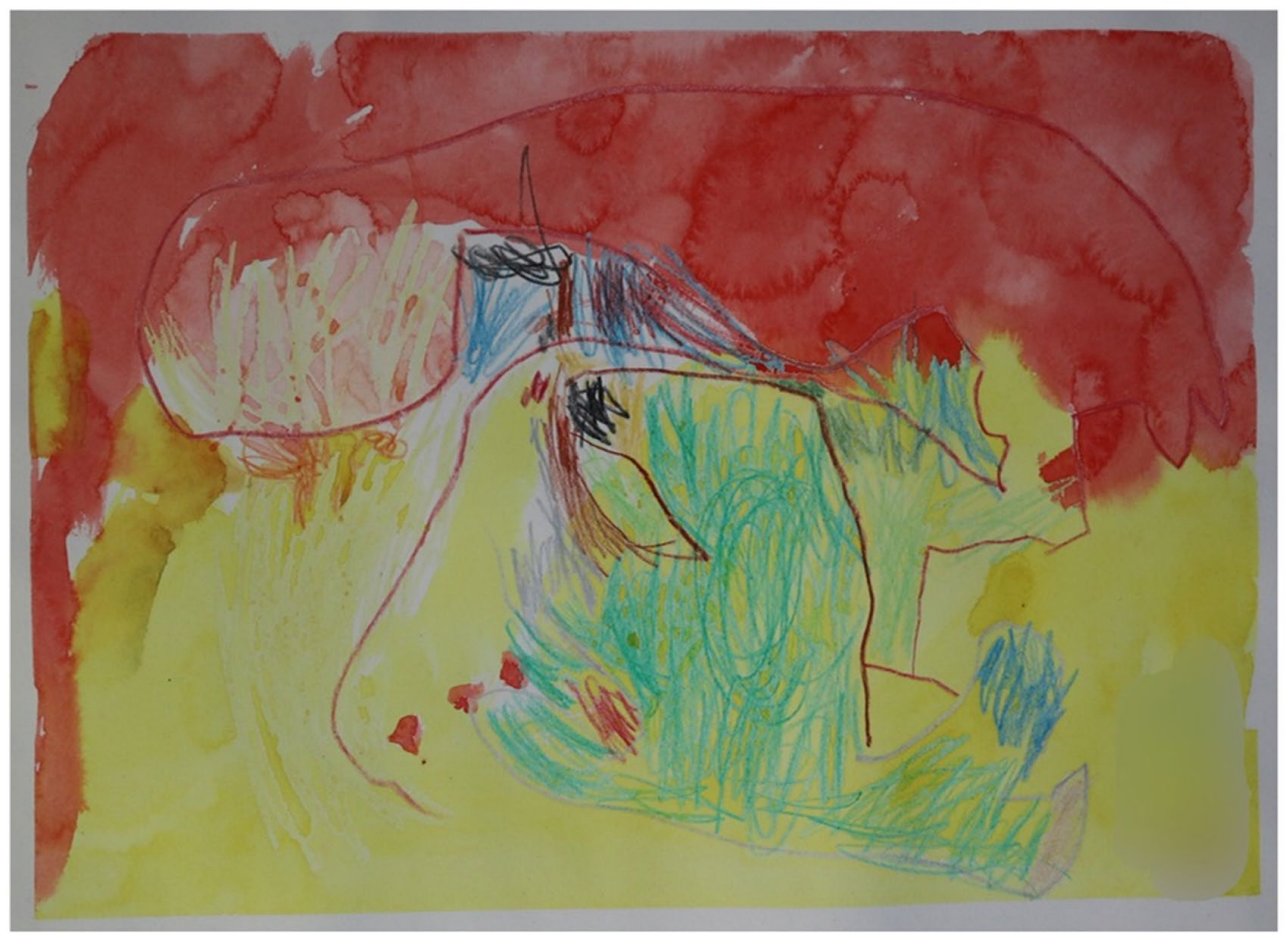
Parents invariably seemed proud of their children’s creations and encouraged them to show and give them to others, like the father of a 4-year old boy who asked his son multiple times if he could have the painting as a birthday gift. The right hand bottom corner has been blurred for the purpose of anonymity, as the child had written the first letter of his name, and his age. Aquarelle over bees wax crayon.

### 2.10 The child achieves something beyond its limits – a triumph

Within the secure environment fostered by art therapy, children engaged in creative expression, facilitating the emergence of their healthy inner child. In the midst of the body failing and so much of them and their life being stripped away, the children’s achievements in art therapy often presented a striking contrast to their outwardly apparent conditions. In other words, there was a marked contrast between the exterior sick self, and the inner healthy self. Curiously, it seemed as if all the children – even as small as 3 years old, and even those who were terribly affected by their illness and treatment – had a clear idea of finishing the painting. In other words, they knew if it was complete or not, and there were not even remote signs of giving up until it was done. The girl who created a pop-up card in her art therapy session relaxed notably after she and the therapist managed to assemble the card and it worked. The 6-year old girl who is halted by the adults during her second painting was clearly not happy with this decision, and began ignoring the therapist and her father when they tried to talk to her. She wasn’t satisfied until the therapist agreed to come back later the same day. Pushing through in this manner, often going well beyond what one might expect from these children, seemed nothing less than a triumph. This feeling seemed to be shared by the children, who beamed with pride, as did their parents. The observational data captured within this theme illustrate poignant moments involving some of the most critically ill children and their journeys toward creating unexpectedly remarkable artwork. Additionally, these observations highlight moments of pride exhibited by both the children and their parents upon the completion of the art projects, showcasing the profound impact of art therapy in facilitating a sense of achievement and resilience among young patients.

There was a stark contrast between some of these children’s physical conditions and what they eventually ended up creating:

In the room is a 12 year old girl, lying in bed. She is wearing a hospital gown. She has a blanket under one of her legs, and the other leg crossed over it. She is entirely passive, and does not move a muscle. She has a sad demeanor. [The therapist talks to the child and tries to engage her from time to time.] The child doesn’t answer and doesn’t move, only occasionally moves the eyes. [The therapist attaches an easel to the table such that the child can paint while lying on her back.] The child is pale. She has a flat facial expression.

**Figure 3:**
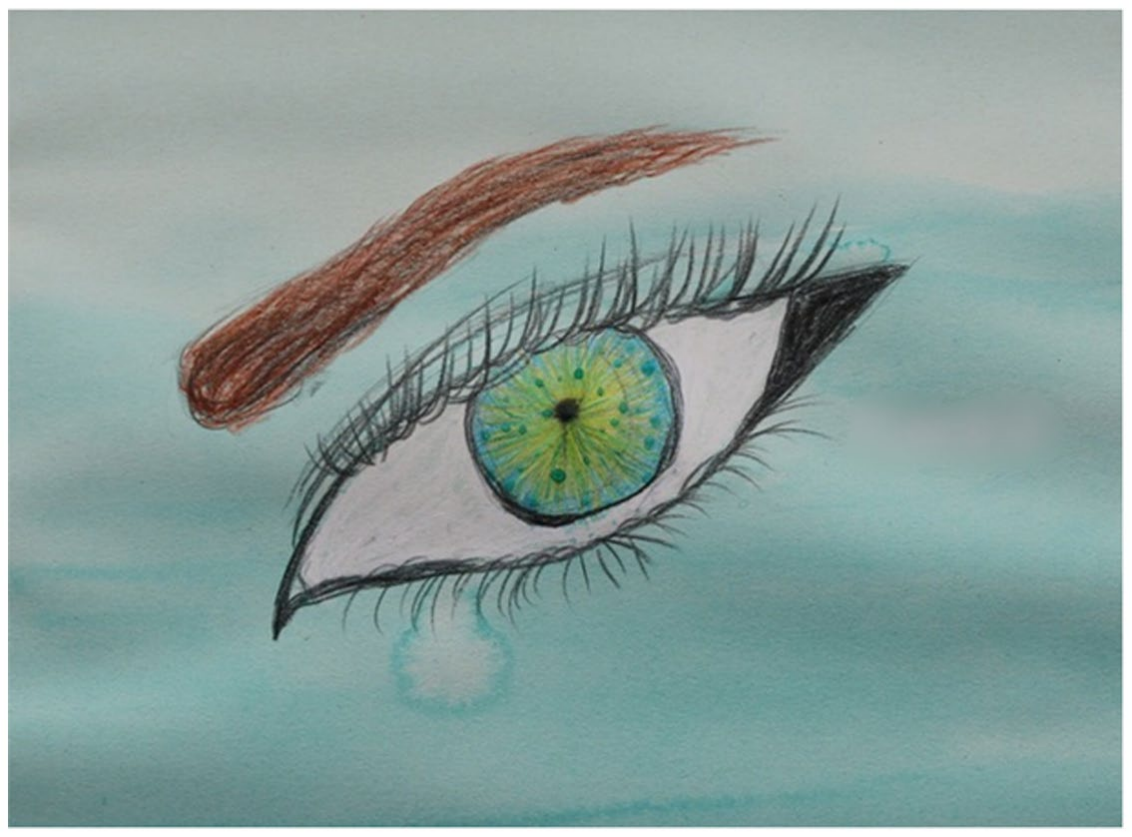
One 12-year old girl moved nothing but her eyes and one hand, but made a beautiful, strong painting. Middle right hand side has been a blurred for the purpose of anonymity, as the child had written her name. 30 × 42 cm beeswax and aquarelle.

Despite being heavily reduced this child goes on to produce a lovely, strong painting (see Figure 3).

One 11-year old child had paintings on the wall of her hospital room from previous art therapy sessions that indicated she normally had a strong grip, used big colors and had good technique. However, in this session she was almost absent, looking down and not answering anybody. She was thin and pale, and next to her wheelchair was a drip stand. She did not react to anything happening in the room, and ignored any attempts at communication. From time to time she cleared her throat so violently it sounded like she might throw up. She seemed on the verge of tears. There was a feeling of a child pressed to the limits. When the therapist was finished prepping the table, though, the child lifted an orange crayon:

She begins to draw from the right bottom corner. She has a weak grip but some energy; she fills the corner with a tight color. She looks passively at the paper but is a bit more present. Then she continues with a reddish brown outside the orange. She begins drawing without looking at the paper; she looks away at the paintbrushes. ‘That crayon you’ve got—’ the therapist begins to say. ‘Krrj!’ The child clears her throat. She has to wipe her mouth, and then puts the crayon down. The room becomes silent.

‘How about the magical one?’ the therapist suddenly says. ‘No, that one’s over here’ she adds, mostly to herself. She gets it from the art trolley and puts it on the table in front of the child. The child takes the white crayon immediately. She draws a big, strong shape with circular motions in the middle of the paper. She suddenly seems to have some force in her hand. Her eyes are clear. ‘Yes, good, use some force!’ the therapist cheers her on.

It becomes silent. The child has put the crayon down and rests her head in her hands. ‘Maybe fill it with color, for example?’ the therapist suggests carefully ‘—somewhere… or… have different things in there…?’ The child doesn’t respond.

The therapist is looking for something on her trolley. The child leans her head on her hand, and partly covers her face. ‘I have– I have the regular colors, right?’ the therapist says, ‘And then I have– Well, but then I hadn’t filled those up…’ She is absent-mindedly talking to herself and lifts up a rack with small glass jars with different colors. ‘This one is a bit– uh, brownish yellow.’ She begins introducing the different colors when the child suddenly with a clear and firm voice says: ‘Purple.’

The child is now painting with long, steady brushstrokes, from the left hand corner and all the way over to the right. You can tell that she has done it before, that she knows the technique. She doesn’t say anything, but is following closely.

For this child, in what undoubtedly was a difficult session, the completion of her painting seemed a particularly strong accomplishment (Figure 5). She was exhausted afterwards and went straight to bed without speaking to anyone or taking part in the painting being hung up. Again, this girl’s painting gave no hint at how severely affected she was.

**Figure 4:**
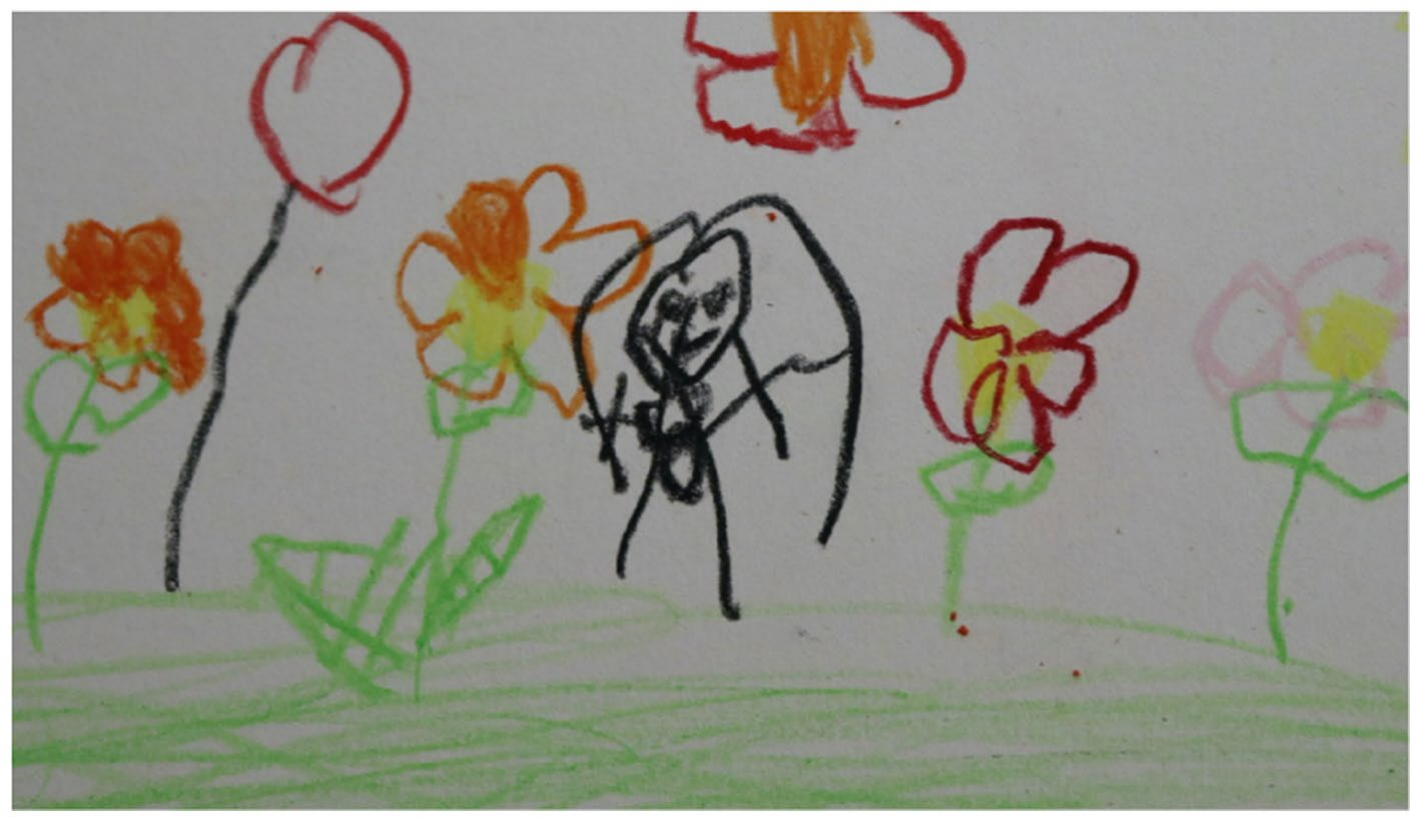
Detail from a drawing by a 6-year old girl, who has drawn her mother in the middle of a colorful garden. The mother isn’t currently with her in the room, and the girl says the drawing will be a gift for her mother. Beeswax crayon.

Most children seemed impressed with their own accomplishment, like this 8-year old girl:

‘Now we’re done’ the therapist says, ‘There! What do you think?’ ‘*Really* nice!’ the child says and smiles. She straightens her back.

The children gladly let their painting be hung on the wall for all to see, like this 12-year old boy who wanted to hang his painting where everyone could see it:

‘There’ the therapist says, ‘And now– Perhaps you want it–’ She holds the painting up to the wall to show what it might look like hanging there. ‘Here, on the wall?’ she says, ‘Here, right? Then you’ve got something to look at? Isn’t that nice?’ ‘Yes’ the child says, ‘What I really want is to hang it *on the door!*’ ‘Oh!’ the therapist says, ‘That’s– That’s really what I wanted to ask you.’

A majority of the children said they wanted to give their piece of art to a significant person in their life; parents, siblings and an uncle were mentioned. This 6-year old girl was struggling to speak, and her mother was not with her in the hospital room (Figure 4):

‘What can you tell me about your painting?’ I ask. (…) ‘I– thought– it was– really nice– and– I– made– it– for Mom’ the child says.

The parents invariably seemed proud of their children’s creations and encouraged them to show or give them to others, including themself (see Figure 2):

‘Maybe it’s a birthday present for me, since it’s my birthday today?’ says the father. (…) The therapist looks at the child. ‘Oh wow, is it Dad’s birthday today?’ she says happily. ‘Is it a birthday gift for me, [Name]? The beautiful painting?’ the father tries again, but the child doesn’t seem to listen. ‘Maybe?’ says the father.

For this child, in what undoubtedly was a difficult session, the completion of her painting seemed a particularly strong accomplishment. She was exhausted afterwards and went straight to bed without speaking to anyone or taking part in the painting being hung up. Again, like in the example of the child where the end product was in stark contrast with her physical condition, this girl’s painting gave no hint at how severely affected she was.

**Figure 5:**
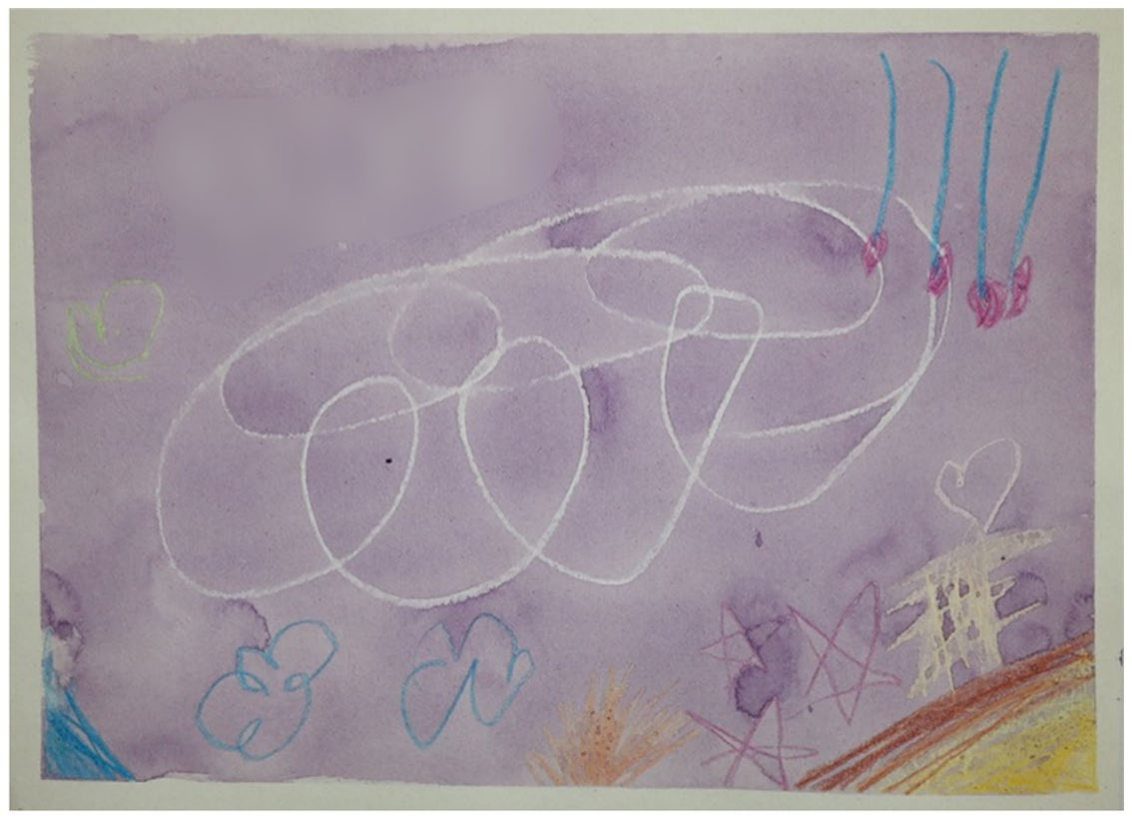
For this heavily reduced 11-year old child, the end result was nothing short of a triumph. The top left-middle has been blurred for the purpose of anonymity, as the child had written her name. Note that after her name, she added four exclamation marks. 21 × 30 cm aqaurelle over beeswax crayons.

## 3 Discussion

**Figure 6:**
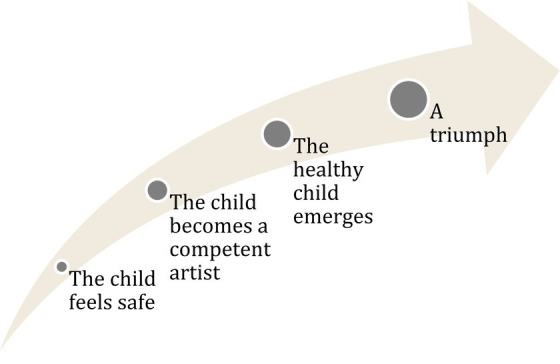
The art therapist fosters a trusting relationship with the child, which serves to awaken creative competencies. This nurturing environment enables the healthy aspects of the child’s personality to surface. The culmination of this process is described as a significant triumph.

An art therapist introduces a sanctuary of creativity in a disorienting children’s ward where illness overshadows normalcy. She quickly establishes trust, making the children feel seen and in control. She adjusts to each child’s capability by teaching basic art techniques, and shifting tasks to maintain focus. This diverts their attention from pain and discomfort, foregrounding their creative goals. The result is therapeutic: a triumph in art, a tangible achievement to share, and a strengthened bond that functions as a psychotherapeutic alliance (Figure 6).

The parents come across as exhausted, confused and frightened. They seemed desperate for positive moments, and for something concrete they can do to help their child. For the brief duration of the art therapy session, the child - and sometimes even the parent – had a ‘safe’ place for conversation, as they chatted about where in nature aquarelle pigment was found, or what the painting made them think of. The parents seemed happy to see their children happy, and they were relieved to be given concrete tasks.

The findings of this study resonate with the positive effects observed in previous art therapy research. However, these effects are multifaceted and intertwined, making it challenging to isolate and delineate causal relationships one by one. Nevertheless, for initially anxious children, a noticeable shift toward happiness and contentment was apparent. Some children even reported that art therapy made them feel as if they were thinking of “nothing,” which related to their diminished perception of pain and discomfort, suggesting a potential interconnection. This this aligns with previous research in the field by Gürcan and Atay Turan (2021), Shella (2018), Stinley et al. (2015), Madden et al. (2010), and Favara-Scacco et al. (2001).

Furthermore, art therapy provided an alternative to more passive forms of entertainment like iPads and phones. Even children with limited mobility exhibited increased activity and energy throughout the sessions. Their engagement with imagination and playful expressions mirrored other research on positive effects of play within a hospital setting, as evidenced by studies such as Godino-Iáñez et al. (2020).

It is widely acknowledged in the field of art therapy research that the act of creating art itself is inherently enjoyable and serves as a means for children to temporarily set aside their illness and injuries (Aguilar, 2017; Clapp et al., 2019). This enjoyment is underpinned by a burst of creativity that arises when imagination and spontaneous thinking are given free rein. It aligns with the concept of a “flow state” from cognitive psychology, as described by Csikszentmihalyi (2000), where clear goals, immediate feedback, a balance between skills and challenges, and freedom from the fear of failure engender a state of immersion and autotelic work. This process is believed to release dopamine, resulting in a pleasurable experience (Chermahini and Hommel, 2010; Zabelina et al., 2016). In the art therapy sessions of this study, children exhibited excitement and eagerness to begin after witnessing the art therapist’s drawings and paintings, which often featured fantastical elements, granting the children permission to imagine freely. Throughout the session, the art therapist expertly maintained the children’s concentration by intuitively adjusting tasks as necessary. Each task offered immediate, visually satisfying feedback, reinforcing engagement and potentially triggering creativity.

Another vital component of art therapy in this context was the acquisition of artistic techniques. Art therapist Robert Ault (Ault et al., 1988) emphasized the importance of creating a structured process that includes the proper use of materials. This approach not only fosters mastery but also promotes self-esteem and self-efficacy. The art therapist’s teaching style in this study resonates with Vygotsky’s concept of “scaffolding” (Verenikina, 2008), where the therapist gauged the child’s current level of artistic development and provided guidance tailored to their emerging skills. This approach led the children to believe in their capacity to master the techniques, ultimately resulting in successful and completed artworks.

Moreover, the children in this study were granted a high degree of control over their art therapy experience. This approach aligns with art therapist Malchiodi’s (2012) assertion that providing children with autonomy and dignity in an environment largely beyond their control can be profoundly therapeutic. The children made choices regarding the duration of the session, their artistic subjects, materials, colors, and whether they wanted to paint alone, with a parent, or with the art therapist. This sense of control was well-received, as evidenced by their active engagement in decision-making.

One of the most prominent effects of art therapy in hospital settings is its ability to facilitate communication. While this study did not prescribe specific topics for artistic expression, it did uncover spontaneous messages within the artwork. For instance, a 4-year-old non-verbal boy’s vibrant painting suggested he was coping well despite his inability to communicate verbally. Similarly, an 11-year-old girl’s artwork, which depicted toys received during medical procedures, indicated the significance of comfort objects and her own resilience. These revelations align with the concept of “redemption” in narrative identity theories, signifying the capacity to learn and grow through adversity (Adler et al., 2015).

**Figure 7:**
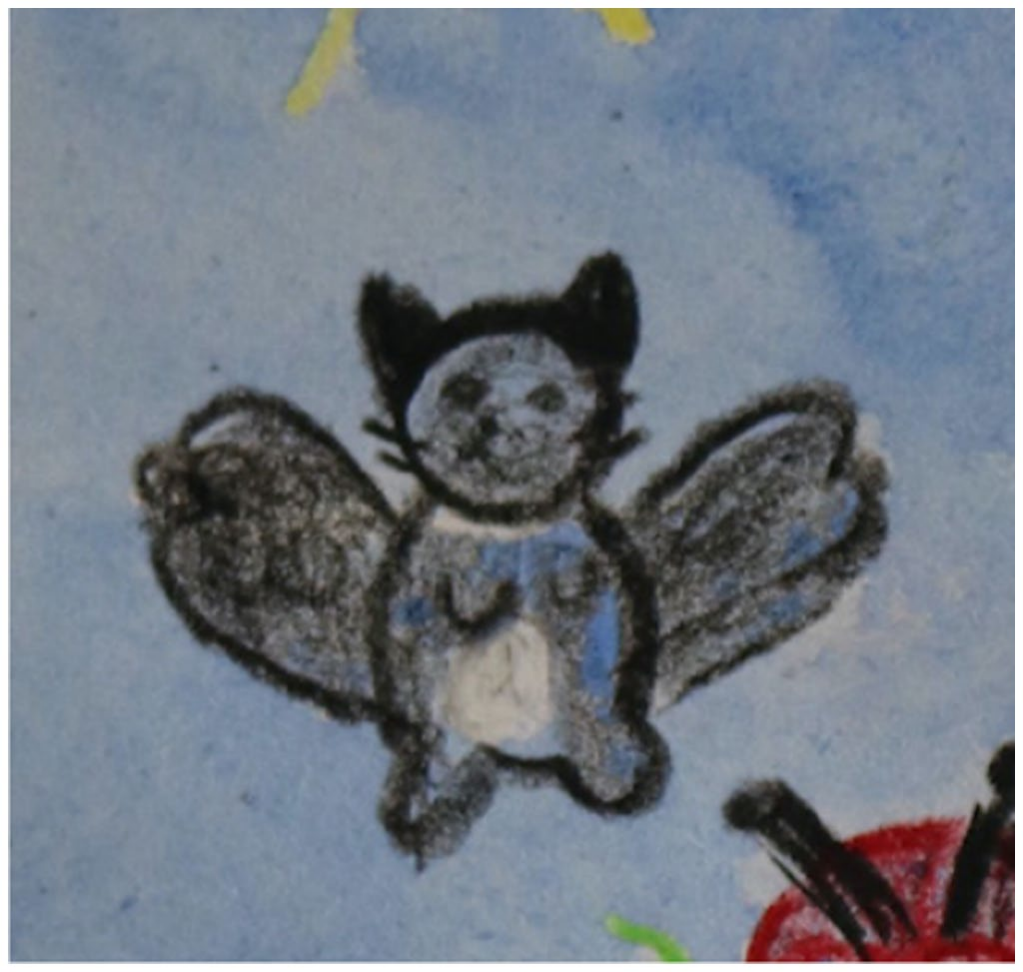
Detail from drawing: An 11-year old girl draws her pet cat, which she says she misses. The cat has wings and is flying in the picture. Aqaurelle over beeswax crayon.

The encouragement of free expression frequently brought forth positive emotions in the children, as they depicted joyous moments with family members, beautiful places, superheroes, and beloved pets (Figure 7). This tendency mirrors findings in research by Nesbitt and Tabatt-Haussman (2008), Madden et al. (2010), and Cowell et al. (2011).

In these art therapy sessions, children were equipped with the tools, knowledge, and freedom to paint whatever their hearts desired. The opportunity to express themselves emerged as a powerful active ingredient for physically limited children. Giving the youngest children choices to increase a sense of control is noted to have improved compliance and reduce the need for sedation in hospitals (Hilton, 2014; Lambert et al., 2014). Phenomenological psychology underscores the significance of the body as the locus of existence and experience (Merleau-Ponty, 1945). For seriously ill children, whose bodily abilities may be severely compromised, the act of creating art can transcend physical limitations. A paintbrush or crayon becomes an extension of intention, a means to experience and communicate with the world, and a reminder of the vibrant person residing within the injured body. This effect aligns with art therapist Kleiven’s (2022) assertion that art therapy allows the healthy child within to emerge, particularly for highly immobile children.

Art-making within the hospital environment may bolster a positive self-image and a sense of identity. Children being referred to as “artists” and receiving input fosters a sense of equality and respect that diverges from the medical focus on illness and injury. The art therapist consulted the children on everything from brush sizes to shades of colors, the same way one might expect from a conversation with a revered colleague. This positive regard, a foundational concept in psychotherapy for over 70 years (Farber et al., 2018), likely contributes to the children’s satisfaction and pride in their artistic endeavors. The act of hanging their paintings for others to see or contemplating gifting them to loved ones engenders feelings of accomplishment and contentment, driven by the release of dopamine and serotonin (Hanson, 2009). Additionally, the positive feedback and support from the art therapist, parents, and even nurses reinforce their sense of competence and belonging within the social group, influencing their self-evaluation and mental well-being (Tsang et al., 2012).

A key element of art therapy for the children in this study was the therapeutic alliance formed with the art therapist. While this concept is fundamental in psychotherapy, it has received less attention in the art therapy research field. The interaction between the art therapist and the children was marked by close turn-taking, mirroring of tone and language, and a profound warmth and respect that communicated genuine care. The children were empowered to exercise control over the artistic process, and the art therapist adeptly interpreted their intentions, fostering a sense of shared purpose and safety. These elements align with the core principles of a therapeutic alliance, which is recognized in psychology as a crucial factor in therapeutic outcomes (Horvath and Luborsky, 1993; Nienhuis et al., 2018).

Interestingly, with some children, there appeared to be moments of tension where they seemingly bullied the art therapist. These behaviors were observed in children with severe, disfiguring illnesses who had endured lengthy treatments. Such behavior resonates with control-mastery theory in psychology (Foreman, 1996), suggesting that patients may reenact negative or traumatic experiences in a safe therapeutic relationship. The children’s actions may have stemmed from feelings of powerlessness, as they tested whether the therapist would respond with inferiority or resilience. Notably, the art therapist’s neutral, supportive response eventually led to a cessation of these behaviors. In one case, the child visibly relaxed after the art therapist assisted in achieving her artistic project, potentially interpreting it as a tangible sign of goodwill. Other children sought to impress the art therapist, an attempt to gauge her response and confirm her positive regard. These dynamics suggest that art therapy can take on attributes akin to psychotherapy when a strong therapeutic alliance is established.

Hospitalization of children imposes immense stress on parents, who play a pivotal role in their children’s mental well-being. The art therapy sessions in this study provided parents with a welcome respite from the medical discussions, offering them a “safe” topic for conversation and a chance to observe their child’s progress. Some parents even took a moment to relax with their phones. This potential role of parental involvement in facilitating successful art therapy for children should not be underestimated.

### 3.1 Scope and limitations

This study is an attempt to delve deep into the world of art therapy for children facing serious somatic illnesses and injuries. While it provides an intimate glimpse into the experiences of younger children, it does not encompass the very youngest (0–3 years old). The study’s limitations include a small sample size, making generalization challenging, as it focused on a single art therapist and her specific approach. Nonetheless, this study aims at rich and detailed descriptions of the research context and participants, allowing for potential transference to other contexts. A notable limitation of this study is its exclusive focus on the children’s subjective experiences of art therapy, thereby omitting pertinent psychological and art-theoretical considerations that could also enrich the understanding of art therapy’s efficacy. Consequently, this study underscores the necessity for further varied explorations of the hospitalization of children and their experiences with art therapy, shedding light on the key elements that contribute to its success.

## 4 Conclusion

The findings of this study underscore the multifaceted benefits of art therapy, from sparking creativity and teaching techniques to fostering autonomy and forming therapeutic alliances, all of which serve to enhance the well-being of young patients in a challenging somatic pediatric setting. A key finding in this study, which has not been devoted much attention so far withing the art therapy field, is that a therapeutic alliance between art therapist and child will emerge, which can facilitate the art making process.

## Data availability statement

The datasets presented in this article are not readily available because the data are not publicly available due to their containing information that could compromise the privacy of research participants. Requests to access the datasets should be directed to PL, ploreskar@gmail.com.

## Ethics statement

The studies involving humans were approved by Regional Committees for Medical and Health Research Ethics. The studies were conducted in accordance with the local legislation and institutional requirements. Written informed consent for participation in this study was provided by the participants’ legal guardians/next of kin. Written informed consent was obtained from the minor(s)’ legal guardian/next of kin for the publication of any potentially identifiable images or data included in this article.

## Author contributions

PL: Writing – original draft, Writing – review & editing. P-EB: Supervision, Writing – review & editing.

## Footnotes

^1^ https://www.forskningsetikk.no/en/

^2^ https://sikt.no/en/about-sikt

^3^ https://helse-bergen.no/om-oss/klinisk-etikk-komit
